# Effect of Oxidative Stress on Extracellular Vesicles Secreted by Caruncular Epithelial Cells Isolated from Bovine Placenta During Pregnancy—Preliminary Results

**DOI:** 10.3390/ani16111717

**Published:** 2026-06-04

**Authors:** Monika Jamioł, Jasmin Galli, Mariusz P. Kowalewski, Jacek Wawrzykowski, Marta Kankofer

**Affiliations:** 1 Department of Biochemistry, Faculty of Veterinary Medicine, University of Life Sciences in Lublin, 20-033 Lublin, Poland; monika.jamiol@up.edu.pl (M.J.); jacek.wawrzykowski@up.edu.pl (J.W.); 2 Institute of Veterinary Anatomy, Vetsuisse Faculty, University of Zurich, Winterthurerstrasse 260, 8057 Zurich, Switzerland; jasmin.galli@uzh.ch (J.G.); kowalewski@vetanat.uzh.ch (M.P.K.)

**Keywords:** cows, pregnancy, placenta, extracellular vesicles

## Abstract

Maintaining the pro- and antioxidative balance during pregnancy is crucial. Oxidative stress (OS) can complicate pregnancy and disrupt maternal–foetal communication, which is partly mediated by extracellular vesicles (EVs). This study investigated how OS affects EV secretion by bovine caruncular epithelial cells isolated from the established bovine placenta (4th month of gestation). To induce OS, cells were treated for 48 h with 1% cigarette smoke extract (CSE), known to increase reactive oxygen species (ROS) levels, while controls remained untreated. EVs were isolated via ultracentrifugation (UC) and size-exclusion chromatography (SEC) and characterized using Tunable Resistive Pulse Sensing (TRPS) technology, transmission electron microscopy (TEM), and Western blotting (WB). WB confirmed small-EV enrichment in fractions 5–8 of SEC by detecting CD9 and flotillin-1 markers. TRPS analysis demonstrated that OS increased EV concentration. In fractions 5–6, CSE treatment increased EV concentration from 1.79 × 10^9^ (mode diameter 127 nm) to 3.12 × 10^9^ particles/mL (122 nm). In fractions 7–8 of SEC, concentration rose from 4.48 × 10^10^ (84 nm) to 7.53 × 10^10^ particles/mL (80 nm). These data indicate that OS increases EV secretion by bovine maternal epithelial cells, providing a basis for future studies on the potential role of stress-induced EVs in foetal–maternal communication.

## Simple Summary

A successful pregnancy in dairy cows is essential for both animal welfare and milk production. However, the high physical demands of producing milk put the cow’s body under severe stress, which can harm the developing calf. The communication between the mother and the fetus involves small cellular packages called extracellular vesicles. Our study aimed to understand how oxidative stress affects the release of these messenger packages from the mother’s placental cells. Using a laboratory model, we exposed cow maternal placental cells to a cigarette smoke extract to mimic severe cellular stress. We discovered that this exposure not only harmed the cells but also caused them to release a markedly increased number of these vesicles. This significant overproduction suggests that stressed cells may release these vesicles as part of a broader cellular stress response, possibly involving intercellular signaling or removal of damaged cellular components. Understanding this communication system helps explain why pregnancies fail in stressed farm animals, paving the way for better veterinary care, improved dairy farming efficiency, and enhanced animal welfare.

## 1. Introduction

The establishment and maintenance of a successful pregnancy in dairy cattle is a complex, highly coordinated process that relies on functional communication between the maternal and foetal compartments. The bovine placenta, characterized by its cotyledonary and synepitheliochorial nature, facilitates this critical interaction primarily through specialized structures known as placentomes, formed by the interdigitation of foetal cotyledons and maternal caruncles [1,2]. At the foeto–maternal interface, the caruncular epithelium acts as a critical regulatory barrier, playing an indispensable role in nutrient exchange, immune modulation, and endocrine signaling [3,4].

In recent years, small extracellular vesicles (EVs), including exosomes, have emerged as pivotal mediators of intercellular communication within the reproductive tract. These nano-sized, lipid-bilayer-enclosed particles, typically ranging from 30 to 150 nm in diameter, are actively secreted by different cell types and carry a complex, highly specific cargo of proteins, lipids, and nucleic acids [5]. In the bovine foeto–maternal interface, the release of EVs is recognized as a fundamental mechanism of bidirectional cross-talk. While foetal-derived EVs have gained considerable attention, with binucleate trophoblast cells releasing exosome-like vesicles to modulate maternal receptivity [6,7], research has also highlighted the essential role of the maternal secretome. Specifically, maternal endometrial epithelial cells secrete exosomes that transport regulatory molecules, such as miR-218, to support proper trophoblast development [8]. Furthermore, placental exosomes exhibit unique biochemical features, such as the presence of surface-associated polysialic acid, which modulates their biological interactions within the uterine environment [9]. Such vesicles often carry specialized proteins like the fusogenic syncytin BERV-K1, which is critical for successful foeto–maternal communication [10].

Importantly, the secretion profile and molecular composition of EVs are highly dynamic and profoundly influenced by the physiological and pathological state of the parent cell [11]. Consequently, EVs are not merely passive cellular byproducts but active, stress-responsive vectors that reflect the cellular microenvironment, making them particularly valuable targets for investigating how placental cells adapt to or are impaired by external stressors.

It is well established that the intense metabolic demands of high-yielding dairy cows can naturally disrupt the oxidant–antioxidant balance, leaving reproductive tissues particularly vulnerable to oxidative stress, especially during the demanding periparturient period [12]. As has been noted, the overlapping physiological stress associated with high milk production and concurrent pregnancy keeps dairy cattle in a state of heightened systemic oxidative stress, making the animals’ immune and reproductive tissues highly susceptible to cellular damage [13,14]. When this natural metabolic burden is compounded by exposure to toxic substances present in the environment, the consequences for placental function can be severe.

Cigarette smoke is a well-documented source of toxic chemicals that have been linked to placental dysfunction, increased oxidative stress, and adverse pregnancy outcomes in various mammalian models [15]. Although cigarette smoke exposure does not represent a typical physiological condition in cattle reproduction, it may occur incidentally through passive environmental exposure. More importantly, in the present study, CSE was used as a complex exogenous oxidative stress model rather than as a direct reproduction of natural cattle exposure. Unlike single-component oxidative stress inducers such as H_2_O_2_, CSE contains a mixture of reactive and toxic compounds capable of disturbing cellular redox homeostasis and inducing oxidative damage. In this context, CSE is commonly used across various mammalian in vitro models, including bovine epithelial and endothelial cells, as an inducer of exogenous oxidative stress to study redox-dependent cellular responses [16,17].

While the direct cytotoxicity of CSE and its ability to induce intracellular oxidative stress in placental cells are well established [18], its impact on intercellular communication—specifically the secretion and size profile of maternal EVs—remains largely unexplored. Understanding how such exogenous oxidative stressors alter EV-mediated foeto–maternal communication is crucial for unraveling the mechanisms underlying placenta-related pathologies.

Therefore, this preliminary study aimed to determine whether oxidative stress affects the size and concentration of extracellular vesicles secreted by maternal epithelial cells isolated from the fully established bovine placenta.

## 2. Materials and Methods

### 2.1. Cell Culture

Primary bovine epithelial cells, originally isolated from the caruncles of a cow in the fourth month of pregnancy, were used in the present study. The detailed protocol regarding the tissue collection, cell isolation, and characterization of this cell model has been described previously by Jamioł et al. [19]. The cells were routinely cultured in a full supplemented medium (FSM) consisting of Dulbecco’s Modified Eagle Medium/Nutrient Mixture F-12 (DMEM/F-12 50:50; 15-090-CV, Corning, Corning, NY, USA), supplemented with 10% fetal bovine serum (FBS; 35-079-CV, Corning, Corning, NY, USA), 100 IU/mL penicillin and 100 µg/mL streptomycin (30-002-CI, Corning, Corning, NY, USA), and 2 mM L-glutamine (G7513, Sigma-Aldrich, St. Louis, MO, USA). The cell cultures were maintained in a humidified incubator at 37 °C with an atmosphere containing 5% CO_2_. For all experiments described herein, the cells were used between passages 8–11. Mycoplasma contamination testing was not performed in the present study.

### 2.2. Preparation of Cigarette Smoke Extract (CSE)

Cigarette smoke extract (CSE) was prepared based on a previously described protocol [20], with minor modifications. Briefly, the mainstream smoke from two commercial cigarettes (Marlboro Gold, Philip Morris, Kraków, Poland) was continuously bubbled through 8 mL of phosphate-buffered saline (PBS) contained in a 15 mL conical tube (GenoPlast Biotech S.A., Rokocin, Poland). The extraction apparatus consisted of a 50 mL syringe connected to a 3-way stopcock and extension tubing, with the cigarette tightly fitted into a 1000 µL pipette tip (NEST, Wuxi, Jiangsu, China). For each puff, the syringe plunger was drawn to the 50 mL mark over a 10 s period. This procedure was repeated until it burned down to the filter. The resulting smoke suspension was immediately sterile-filtered through a 0.2 µm pore size PES membrane filter (Biologix Group Ltd., Jinan, China). Based on the modified preparation protocol used in this study, this filtered solution was considered a 50% CSE stock solution. To obtain the final working concentration corresponding to 1% CSE, 300 µL of freshly prepared 50% CSE stock solution was added to serum-free supplemented medium (SM) to a final volume of 15 mL. In the corresponding control group, 300 µL of sterile PBS was added to SM instead of CSE. The final concentration of 1% CSE was selected based on preliminary optimization experiments, in which H_2_O_2_ caused extensive cell detachment, whereas 1% CSE induced measurable oxidative stress while preserving a sufficient adherent cell population for subsequent EV isolation. The CSE was always prepared fresh immediately prior to each experiment. The entire process, from the initiation of smoke extraction to the final application of the 1% CSE to the cell cultures, was strictly controlled and completed within approximately 20 min. This time refers to the preparation-to-application interval and not to the duration of cell treatment.

### 2.3. Cell Viability Assay

Cell viability was evaluated using the MTT assay based on a previously described protocol [21] with minor modifications. Briefly, the cell suspension was seeded into 96-well plates (NEST, Wuxi, Jiangsu, China) at a density of 0.2 × 10^6^ cells/mL (100 µL per well) in full supplemented medium (FSM) and incubated for 24 h at 37 °C in a humidified atmosphere containing 5% CO_2_. Subsequently, the medium was replaced with fresh FSM, and the cells were incubated for an additional 24 h. Following this adaptation period, the medium was aspirated, and the wells were assigned to specific experimental groups, with five replicates per condition (*n* = 5). The cells were then treated with 100 µL of 1% CSE (cigarette smoke extract) in SM (supplemented medium without FBS), pure SM (serving as a vehicle control for CSE), or pure FSM (included as an absolute control to assess the baseline effect of serum deprivation on cell viability). After a 24 h incubation with the treatments, the medium was removed, and 100 µL of MTT (Thiazolyl Blue Tetrazolium Bromide, M5655, Sigma-Aldrich, St. Louis, MO, USA) solution in SM (1 mg/mL) was added to each well. The plates were incubated for 2 h at 37 °C (5% CO_2_). Afterward, the MTT solution was carefully aspirated, and the formed formazan crystals were dissolved by adding 100 µL of dimethyl sulfoxide (DMSO, D2650, Sigma-Aldrich, St. Louis, MO, USA) to each well. The plates were protected from light with aluminum foil and gently mixed on a horizontal microplate shaker (SH30, FINE PCR, Yangchun, Seoul, Republic of Korea) for 5 min. Absorbance was measured at a wavelength of 550 nm using a microplate reader (Labsystems Multiskan RC, Warszawa, Poland). The results were calculated and expressed as the percentage of viable cells relative to the FSM control group. The experiment was performed in two independent replicates.

### 2.4. Intracellular Reactive Oxygen Species (ROS) Detection

The levels of intracellular oxidative stress were evaluated using the CellROX™ Green Reagent (C10444; Thermo Fisher Scientific, Waltham, MA, USA). The cell suspension was seeded into 96-well black plates (ThermoScientific, Rochester, NY, USA) at a density of 0.2 × 10^6^ cells/mL (100 µL per well) in FSM. The timeline for cell adaptation and treatment (initial 24 h incubation, medium replacement, and a subsequent 24 h exposure) was identical to that in the protocol described for the cell viability assay. For this assay, the cells were assigned to four experimental groups: FSM, FSM containing 1% CSE, SM, and SM containing 1% CSE, with four technical replicates per condition (*n* = 4). Following the 24 h treatment period, the medium was aspirated. The cells were then treated with 100 µL of 5 µM CellROX™ Green Reagent diluted in PBS pH = 7.4 (21–040-CV, Corning, Corning, NY, USA) and incubated for 30 min at 37 °C (5% CO_2_). Afterward, the staining solution was removed, and the cells were washed three times with 100 µL of PBS per well to remove the excess, unreacted fluorogenic probe. Finally, 100 µL of fresh PBS was added to each well, and the fluorescence intensity was measured using a VICTOR^3^ 1420 Multilabel Counter fluorescence microplate reader (PerkinElmer, Turku, Finland) at an excitation wavelength of 485 nm and an emission wavelength of 535 nm. The fluorescence values were normalized to the total cell count in each respective well. Immediately after the fluorescence measurement, the cells were trypsinized and counted using an automated cell counter (Countess™ 3 FL, Invitrogen, Life Technologies Corporation, Bothell, WA, USA). The raw fluorescence data were divided by the corresponding cell number, and the final results were expressed as the percentage of relative fluorescence units (RFU) per cell, normalized to the FSM control group. The experiment was performed in two independent replicates.

### 2.5. Extracellular Vesicle (EV) Isolation

Extracellular vesicles were isolated from cell culture media using a combination of differential ultracentrifugation (UC) and size exclusion chromatography (SEC), based on modified protocols previously described by Kowal et al. [22] and Galli et al. [10]. For EV isolation, cells were seeded in 75 cm^2^ flasks and cultured in FSM (37 °C, 5% CO_2_). At approximately 70% confluency, the FSM was carefully removed, and the cells were washed with SM and incubated with 15 mL of pure SM (control group) or SM containing 1% CSE for 48 h. The use of serum-free medium was essential to avoid contamination with EVs originating from FBS. The conditioned medium from four flasks per group was then pooled to obtain one biological sample. The final volume for each pooled sample was approximately 60 mL. In parallel with the medium collection, the cells remaining in the flasks were trypsinized and counted. Cell viability and size distribution were assessed using an automated cell counter (Countess™ 3 FL, Invitrogen, Life Technologies Corporation, Bothell, WA, USA) combined with ReadyCount Green/Red Dead Viability Stain (A49905; Thermo Fisher Scientific, Waltham, MA, USA), which utilizes acridine orange (AO) and propidium iodide (PI) to precisely distinguish between live and dead cell populations. Five experiments from different passages were performed (*n* = 5). The collected medium was supplemented with Halt™ Protease Inhibitor Cocktail (Thermo Fisher Scientific, Waltham, MA, USA) and subjected to sequential centrifugation to remove cells, debris, and large vesicles: 319× *g* (10 min, RT), 1996× *g* (20 min, RT), and 15,120× *g* (20 min, 4 °C). The obtained supernatant was then passed through a 0.22 µm membrane filter and stored at −20 °C for no longer than one month before EV isolation. After being thawed overnight at 4 °C, the samples were ultracentrifuged at 100,000× *g* for 2 h at 4 °C using a Type 70 Ti rotor in an Optima ultracentrifuge (Beckman Coulter, Brea, CA, USA). The resulting EV pellet from each 60 mL sample was resuspended in 0.5 mL of PBS pH = 7.4 (21–040-CVR, Corning) and stored at −20 °C until further processing. Following the initial isolation, the 0.5 mL EV suspension was thawed and loaded onto EV SEC Columns (NBP3-11763, Novus Biologicals, Centennial, CO, USA) and eluted with sterile PBS pH = 7.4. A total of 24 fractions (0.5 mL per fraction) were collected. The EV-enriched fractions were pooled into 1 mL samples (fractions 5–6, 7–8, and 9–10) and subsequently concentrated using Amicon^®^ Ultra-0.5 Centrifugal Filter Units (10 kDa MWCO; UFC5010, Sigma-Aldrich Chemie GmbH, Buchs, Switzerland). Concentration was achieved by centrifugation at 14,000× *g* for 20 min at 4 °C, reducing the sample volume from 1 mL to approximately 50–60 µL. A reverse spin at 1000× *g* for 2 min was then performed to recover the purified EVs. TRPS was performed using freshly isolated EV samples. Samples intended for TEM and Western blotting were stored at −20 °C for no longer than one week, and repeated freeze–thaw cycles were avoided.

### 2.6. Transmission Electron Microscopy (TEM)

The morphology and ultrastructure of the isolated EVs were evaluated using transmission electron microscopy (TEM) (Titan G2 60-300, FEI, Hillsboro, OR, USA). For this analysis, which was performed in two independent biological replicates, a representative specimen was prepared by pooling 20 µL from each of the three concentrated samples (fractions 5–6, 7–8, and 9–10) belonging to the same experimental group (SM control or CSE group). This pooled suspension was then applied onto 200-mesh copper grids coated with a lacey formvar film stabilized with carbon (Ted Pella, Redding, CA, USA). The samples were left to evaporate on filter paper at room temperature. Subsequently, the grids were mounted in a specialized holder and transferred into the electron microscope. Imaging was performed using a Titan G2 60–300 kV high-resolution transmission electron microscope (FEI, Hillsboro, OR, USA) equipped with a field-emission gun (FEG). The microscope was operated at an accelerating voltage of 300 kV. The EV microstructure was visualized in bright-field mode using a Gatan US1000P CCD camera (2k × 2k; Gatan Inc., Pleasanton, CA, USA) as the detector.

### 2.7. Nanoparticle Analysis

The concentration and size distribution of the isolated EVs were measured using Tunable Resistive Pulse Sensing (TRPS) technology with an Exoid instrument (Izon Science, Christchurch, New Zealand). Prior to the analysis, the EV samples were diluted 1:3 (*v*/*v*) in freshly filtered (0.22 µm) PBS. Measurements were performed using an NP150 nanopore (C00921, Izon Science Ltd., Christchurch, New Zealand) stretched to the appropriate baseline current. To ensure accurate quantification and size determination, the system was calibrated immediately before the EV measurements using standard calibration beads (diluted 1:500 in PBS) of known size and concentration (CPC100 Calibration Particles, Izon Science Ltd., Christchurch, New Zealand), according to the manufacturer’s instructions. Data acquisition and analysis were executed using the Exoid Control Suite software (version 1.5.2.0, Izon Science). For each sample, particle blockade signals were recorded at an applied pressure of 2000 Pa and an optimized voltage. Based on these signals, the mean and mode particle sizes, as well as the total particle concentration (particles/mL), were calculated for three independent biological replicates.

### 2.8. Western Blotting Analysis

Western blot analysis was performed in three independent biological replicates according to a previously published protocol [23] with some modifications. Briefly, EV samples were sonicated (2 min, 40 kHz, Intelligent Ultrasonic Generator V6.3) in RIPA lysis buffer (20-188, Millipore, Merck, Darmstadt, Germany) and Halt™ Protease Inhibitor Cocktail (Thermo Fisher Scientific, Waltham, MA, USA). The protein concentration from EV samples was too low to be measured by Bradford or BCA assays; therefore, 33% (*v*/*v*) sample buffer was added to 50–60 µL of sample. For the flotillin-1 detection, the proteins were solubilized in a reducing sample buffer (62.5 mM Tris-HCl, pH = 6.8, 2% SDS, 5% β-mercaptoethanol, 20% glycerol, 0.003% bromophenol blue). For CD9 detection, a non-reducing sample buffer (lacking β-mercaptoethanol) was used, as required for tetraspanins [10]. After the denaturation step (95 °C, 5 min, shaking), the samples were separated by 12.5% SDS-PAGE and transferred to PVDF membranes (pb9320; Invitrogen, Thermo Fisher Scientific, Carlsbad, CA, USA) with Trans-Blot^®^ Turbo™ Transfer System (25V, 25 min, Bio-Rad Laboratories, Inc., Hercules, CA, USA). The membranes were blocked for 1 h in Animal-Free Blocker (SP-5030, Vector Laboratories, Burlingame, CA, USA) diluted to a 1× working concentration in TBST (20 mmol/L Tris, 150 mmol/L NaCl, and 0.1% Tween 20; pH = 7.6) and then incubated overnight at 4 °C with the following primary antibodies: flotillin-1 (1:1000; MA5-50274, Invitrogen, Thermo Fisher Scientific, Carlsbad, CA, USA), and CD9 (1:1000; MA1-19301, Invitrogen, Thermo Fisher Scientific, Carlsbad, CA, USA). Afterwards, the membranes were washed with TBST (3 × 15 min) followed by incubation with secondary antibodies.

Two distinct signal detection methods were employed depending on the target protein.

For the detection of Flotillin-1, the membranes were incubated with a horseradish peroxidase (HRP)-conjugated anti-mouse IgG secondary antibody (1:3750; W402B, Promega, Madison, WI, USA) for 1 h at RT. After washing steps in TBST (5 × 10 min), the protein bands were visualized using the SuperSignal™ West Pico PLUS Chemiluminescent Substrate (34580; Thermo Fisher Scientific, Pierce Biotechnology, Rockford, IL, USA) and imaged with a ChemiDoc^TM^ XRS+ (Bio-Rad, Laboratories, Inc., Hercules, CA, USA).

For the detection of CD9, a biotin–streptavidin amplification system was utilized, followed by colorimetric detection. The membranes were first incubated with a biotinylated goat anti-mouse IgG secondary antibody (1:10,000; 31800, Invitrogen, Thermo Fisher Scientific, Carlsbad, CA, USA) for 2 h at RT. Following washing steps in TBST (3 × 10 min), the membranes were incubated with HRP-conjugated streptavidin (1:20,000; SNN1004, Invitrogen, Thermo Fisher Scientific, Carlsbad, CA, USA). Finally, the CD9 bands were visualized colorimetrically utilizing the DAB Substrate Kit (34065, Invitrogen, Thermo Fisher Scientific, Carlsbad, CA, USA) and were scanned with Epson GT-X980 scanner (Seiko Epson Corporation, Suwa, Japan).

The obtained bands were compared with protein mass standards from 10 to 250 kDa (161-0374; Precision Plus Protein™ Dual Color Standard, Bio-Rad Laboratories, Inc., Hercules, CA, USA). The molecular weights were estimated with the GelAnalyzer software (Version 23.1.1; Istvan Lazar Jr. and Istvan Lazar Sr., Hungary).

### 2.9. Statistical Analysis

Statistical analysis was performed using Statistica software (Version 13.0, StatSoft, Poland, TIBCO Software Inc., Palo Alto, CA, USA). The normality of the data distribution and the homogeneity of variances were assessed using the Shapiro–Wilk and Levene’s tests, respectively. For the MTT assay, differences among multiple experimental groups were evaluated using one-way analysis of variance (ANOVA) followed by Tukey’s HSD post hoc test. CellROX fluorescence data were analyzed using two-way ANOVA with medium and CSE treatment as factors, followed by Tukey’s HSD post hoc test. TRPS-derived particle concentrations and modal particle diameters were analyzed using repeated-measures ANOVA, with treatment as the between-subject factor and SEC fraction as the repeated factor. Particle concentration data were log10-transformed prior to statistical analysis. All data are presented as the mean ± standard deviation (SD). Differences were considered statistically significant at *p* < 0.05.

## 3. Results

### 3.1. The Effect of CSE and Serum Deprivation on Cell Viability

**Figure 1:**
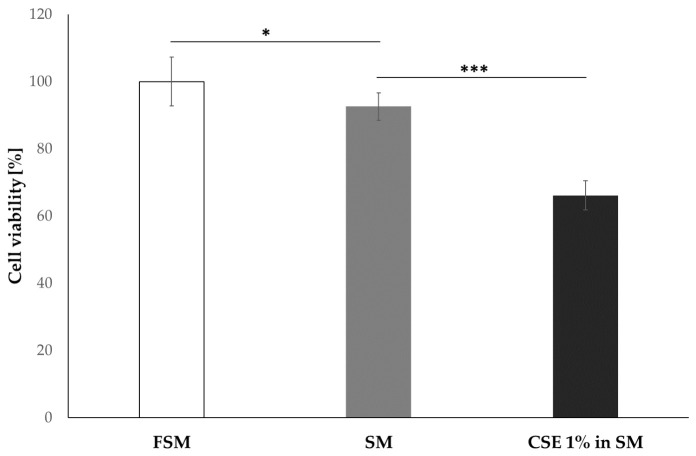
The effect of cigarette smoke extract (CSE) and serum deprivation on cell viability. The cells were exposed to 1% CSE, pure supplemented medium without FBS (SM), or full supplemented medium (FSM) for 24 h. Cell viability was evaluated using the MTT assay and expressed as a percentage relative to the FSM control group (set as 100%). Data are presented as the mean ± SD. Statistical significance was determined using one-way ANOVA followed by Tukey’s HSD post hoc test. * *p* < 0.05 and *** *p* < 0.001 indicate significant differences between indicated experimental groups.

Cell viability was assessed after a 24 h exposure to 1% CSE, with pure SM and FSM serving as controls (Figure 1). The raw absorbance values were normalized to the FSM group, which was set as 100% cell viability. Serum deprivation alone (SM group) caused a slight but significant reduction in cell viability to 92.58% ± 4.07 compared to the FSM control (*p* < 0.05). In contrast, 1% CSE markedly decreased viability to 66.13% ± 4.33 compared to the SM control (*p* < 0.001).

**Figure 2:**
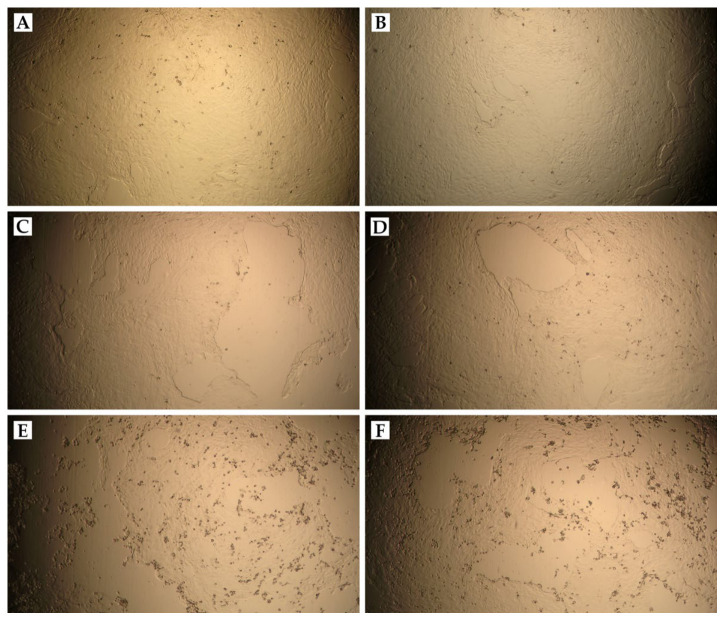
Representative images of bovine caruncular epithelial cells after 24 h exposure to FSM (**A**,**B**), SM (**C**,**D**), or SM containing 1% CSE (**E**,**F**). Images were taken immediately before the MTT assay (light micrographs, 4× magnification).

Representative light microscopy images taken immediately before the MTT assay confirmed visible morphological alterations in CSE-treated cells, including reduced monolayer integrity and an increased number of detached cells compared with FSM and SM controls (Figure 2).

### 3.2. The Effect of CSE and Serum Deprivation on Intracellular ROS Production

**Figure 3:**
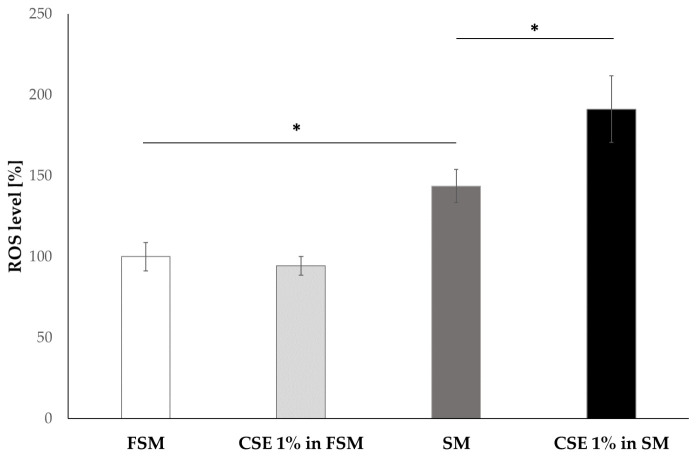
The intracellular reactive oxygen species (ROS) production induced by cigarette smoke extract (CSE) and serum deprivation. Cells were exposed to fully supplemented medium (FSM), FSM containing 1% CSE, supplemented medium without FBS (SM), or SM containing 1% CSE for 24 h. Intracellular oxidative stress was assessed using the CellROX™ Green Reagent. To account for variations in cell density, raw fluorescence values were normalized to the corresponding cell number in each well and expressed as a percentage relative to the FSM control group (set as 100%). Data are presented as the mean ± SD. Statistical significance was determined using two-way ANOVA followed by Tukey’s HSD post hoc test. * *p* < 0.05 indicates significant differences between indicated experimental groups.

Intracellular ROS levels were measured using the CellROX™ Green assay. The raw fluorescence values were normalized to the corresponding cell number in each well and expressed as a percentage relative to the FSM control (100%) (Figure 3). Serum deprivation alone (SM group) induced a marked increase in baseline oxidative stress to 143.6% ± 13.0. Exposure to 1% CSE in serum-free conditions (SM + CSE) further exacerbated ROS production, reaching 191.1% ± 30.3. The administration of 1% CSE in fully supplemented medium (FSM + CSE) did not elevate ROS levels (94.4% ± 1.8%).

### 3.3. Evaluation of Cell Condition Following the EVs Production

**Figure 4:**
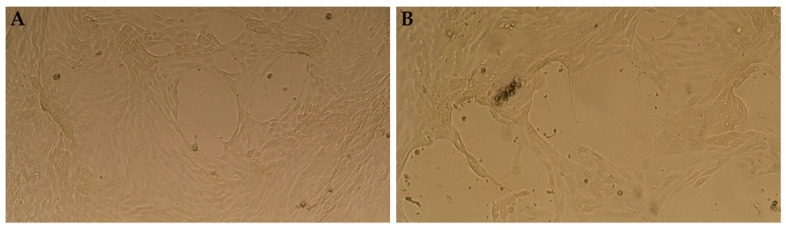
Representative images of bovine caruncular epithelial cells after the 48 h EV-production phase in serum-free medium. Cells were cultured in SM (control; (**A**)) or in SM containing 1% CSE (**B**). Images were taken after conditioned medium collection (light micrographs, 10× magnification).

To ensure the quality of the conditioned medium prior to EV isolation, the cell condition was evaluated after the 48 h EV-production phase in serum-free medium. Representative light microscopy images taken at the end of this phase are shown in Figure 4. Compared with the SM control, CSE-treated cultures appeared to show reduced monolayer coverage.

Automated dual-fluorescence cell counting revealed that the overall morphology, specifically the size of the live cells, remained relatively stable between the groups (15.45 µm in the control vs. 15.65 µm in the CSE-treated group). As expected, dead cells in both groups exhibited a reduced size (12.96 µm and 13.62 µm, respectively), characteristic of apoptotic cell shrinkage. Additionally, in the control group, the live (GFP-positive) cell population was maintained at 96%, with RFP-positive (dead/dying) cells accounting for 35%. Although exposure to 1% CSE decreased the mean proportion of live cells to 84% and correspondingly increased the RFP-positive population to 49%, these differences did not reach statistical significance due to the high variance observed between replicates.

### 3.4. Characterization of EVs

**Figure 5:**
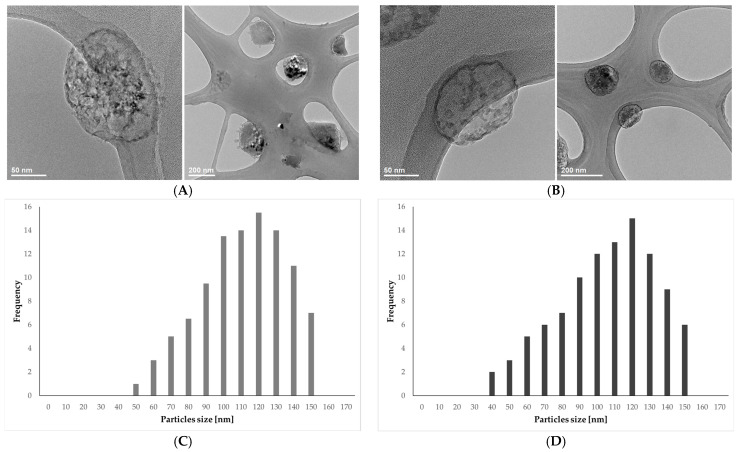
Transmission electron microscopy (TEM) analysis of EVs isolated from cell culture supernatants. TEM images of the control (**A**) and 1% CSE-treated (**B**) samples. Particle size distribution of the pooled fractions (5–10) for the control (**C**) and 1% CSE-treated (**D**) samples.

Transmission electron microscopy (TEM) analysis confirmed the presence of extracellular vesicles in both the control (Figure 5A) and CSE-treated (Figure 5B) samples. The obtained micrographs revealed predominantly spherical particles that maintained structural integrity without widespread aggregation. To quantitatively evaluate these observations, the particle size distribution was determined based on TEM imaging. In the control group, the isolated EVs exhibited a size range from 50 to 150 nm, with a mean diameter of approximately 111 nm and a mode size of 120 nm (Figure 5C). In the CSE-treated samples, the EVs displayed a size distribution ranging from 40 to 150 nm, with a mean vesicle diameter of approximately 106 nm and a mode size of 120 nm (Figure 5D).

Further quantitative analysis of EV size distribution and yield was performed using Tunable Resistive Pulse Sensing (TRPS) for the three independent replicates of EVs isolated from cell culture supernatants (Table 1). Focusing on the main elution peak, corresponding to fractions 7–8, which was highly abundant in vesicles, the control group showed a predominant mode size of 84 nm and a mean ± SD concentration of 4.48 × 10^10^ ± 1.34 × 10^10^ particles/mL. In CSE-treated samples, the corresponding mode size was 80 nm, while particle concentration reached 7.53 × 10^10^ ± 0.21 × 10^10^ particles/mL. Overall, CSE exposure significantly increased EV concentration across SEC fractions (*p* < 0.05), whereas the effect of CSE did not significantly differ between fractions. To account for differences in cell survival after the 48 h EV-production phase, TRPS-derived EV concentration was additionally normalized to the number of viable cells present in the corresponding pooled samples. After normalization, CSE-treated cells still showed higher EV concentration per 10^6^ viable cells compared with the SM control across SEC fractions, although this effect was just above the conventional threshold for statistical significance (*p* = 0.0507). SEC fractions differed significantly in particle concentration (*p* < 0.001), reflecting the distribution of EVs across the elution profile. In contrast, CSE treatment did not significantly affect modal particle diameter, although modal diameter differed significantly between SEC fractions (*p* < 0.001).

**Figure 6:**
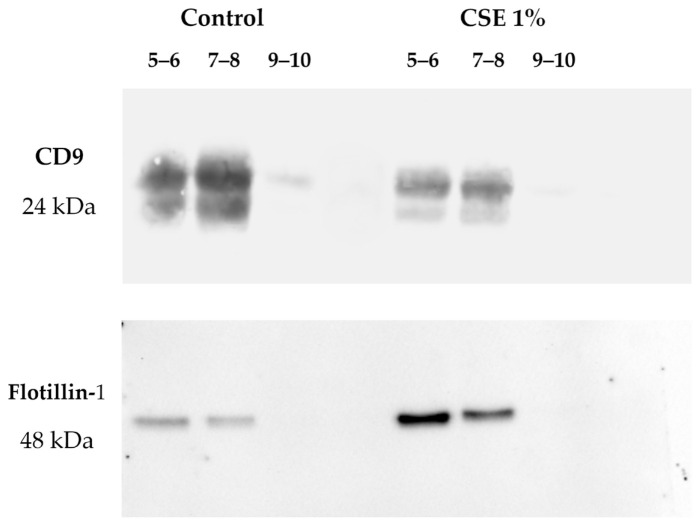
Western blot analysis of EV samples isolated from bovine caruncular epithelial cells (4th month of gestation). Numbers 5–6, 7–8, and 9–10 represent the fractions collected after size exclusion chromatography and subsequent concentration. The original Western blot images are provided in Figures S1 and S2.

WB confirmed EV enrichment in fractions 5–6 and 7–8, showing CD9 (approx. 24 kDa) and Flotillin-1 (approx. 48 kDa) bands (Figure 6) in both the control (SM) and CSE-treated samples.

## 4. Discussion

Establishing a functional connection between the foetus and the mother is crucial for maintaining pregnancy, as it is a dynamic process regulated by constant intercellular communication. In recent years, there has been growing interest in investigating the role of EVs as active factors involved in this communication [24,25]. Although the high metabolic demands of dairy cattle naturally expose their reproductive system to oxidative stress [26,27], the precise impact of external stressors on the maternal vesicular secretome remains to be fully investigated. In the present study, we utilized an in vitro model of bovine caruncular epithelial cells to demonstrate for the first time that exposure to 1% CSE disrupts cellular redox homeostasis and significantly alters the EV secretion profile, driving a marked increase in vesicle release.

To accurately characterize the EV molecules released by caruncular cells, without the interfering influence of serum-derived vesicles, the experiment was conducted strictly under serum-free conditions. Our preliminary viability and ROS assays revealed key baseline dynamics, leading to the conclusion that serum deprivation acts as a mild physiological stressor, causing a slight decrease in cell viability and an increase in baseline oxidative stress. This observation is consistent with established in vitro models, in which the sudden withdrawal of essential growth factors induces mild cellular stress [28,29]. However, the addition of 1% CSE under these serum-free conditions significantly exacerbated this state. The marked increase in intracellular ROS production (an approximate 33% increase relative to the SM control) and the resulting reduction in cell viability (an approximate 28.5% decrease compared to the SM control) reflect the cytotoxicity and free radical damage caused by CSE. This pronounced induction of oxidative stress at the foeto–maternal interface aligns with findings from human placental models. Sidle et al. (2007) demonstrated that treating human trophoblast cells with CSE induces severe oxidative stress, triggering an immediate and compensatory antioxidant response, such as the upregulation of heme oxygenase-1 (HO-1) [30]. Furthermore, Jin et al. (2018) reported that CSE exposure significantly elevates ROS production in primary cells of the human foeto–maternal interface, simultaneously activating the p38MAPK stress pathway and prompting an inflammatory response [31]. While specific data concerning the direct impact of CSE on the bovine placenta are scarce, its detrimental effects have been well-documented in other bovine in vitro models. For instance, Cantral et al. (1995) demonstrated that CSE exposure impairs the attachment and migration capabilities of bovine bronchial epithelial cells [17]. Additionally, Le et al. (2014) showed that CSE induces marked cytotoxicity and significantly decreases endothelial nitric oxide synthase (eNOS) expression in bovine aortic endothelial cells [16].

Importantly, the administration of 1% CSE in fully supplemented medium did not cause an increase in ROS levels. This is not surprising, given that serum supplements are naturally rich in antioxidant enzymes and radical-scavenging proteins, such as albumin, which effectively neutralize oxidative stress in cell culture models [32]. Consequently, our protocol successfully establishes a reliable model of oxidative stress at the cellular level, providing a solid foundation for analyzing the stress-induced cellular response via vesicle release.

Both TEM and TRPS analyses showed that the isolated nanoparticles fell mainly within the typical size range expected for small EVs [33]. Furthermore, Western blotting confirmed the presence of EV markers including CD9, a tetraspanin integral to membrane organization and EV biogenesis [34], and flotillin-1, a lipid raft-associated protein critical for endosomal trafficking [35].

Interestingly, although the ultrastructural morphology and modal size of the vesicles remained unchanged regardless of the culture conditions (with or without CSE), TRPS profiling revealed a significant change in the number of secreted vesicles. Indeed, one of the most important findings of this study is the stress-induced increase in EV release. Importantly, after normalization of EV concentration to the number of viable cells, CSE-treated samples still showed higher EV values across SEC fractions, suggesting that altered cell survival alone is unlikely to fully explain the observed increase in particle concentration. Our analysis revealed a substantial increase in particle concentration following exposure to CSE across SEC fractions, with the highest particle abundance observed in the main peak fractions (7–8). This finding is consistent with previous studies showing that stress conditions can enhance EV release or alter EV-associated cargo in placental and reproductive cell models. For example, severe endoplasmic reticulum stress in BeWo choriocarcinoma cells increased EV release, and antioxidant treatment reduced this response, supporting the link between cellular stress, oxidative imbalance, and EV secretion [36]. In addition, EVs released from stressed endometrial cells have been shown to affect trophoblast responses, further supporting the role of stress-related EV changes in reproductive cell communication [37]. This pronounced quantitative response strongly suggests that increased EV release acts as an adaptive mechanism of the caruncular epithelium in the face of oxidative stress. This interpretation is strongly supported by the results of other studies. As Ryu et al. (2018) comprehensively summarized, CSE-induced excessive EV secretion is a process that is strictly dependent on the redox balance in various cell types [38]. This excessive release is likely caused by redox-dependent thiol modifications and, importantly, can be effectively inhibited by the administration of antioxidants [38]. Evidence suggests that cells may utilize enhanced EV biogenesis as a mechanism for removing oxidized lipids, misfolded proteins, and toxic intracellular metabolites that accumulate during oxidative stress [39]. In this context, increased EV release by maternal placental cells may reflect an adaptive cellular response to CSE-induced oxidative stress. Therefore, the observed increase in EV concentration should be interpreted primarily as a quantitative response of caruncular epithelial cells to CSE-induced stress. Since stress conditions may influence EV cargo and biological activity [40,41], whether these EVs also differ in molecular composition or exert functional effects on other placental cells remains to be determined.

Nevertheless, several methodological aspects should be considered when interpreting these findings. Although the isolated particles showed typical small-EV morphology and expressed selected EV-associated markers, namely CD9 and flotillin-1, additional characterization would be required to determine the precise EV subtype and to further assess sample purity. In addition, oxidative stress was assessed mainly by intracellular ROS production, while additional oxidative damage markers, such as malondialdehyde (MDA), and antioxidant response markers were not evaluated due to the limited amount of available biological material. Moreover, no functional assays were performed to determine whether EVs released under CSE-induced stress affect other placental cells. Finally, mycoplasma contamination testing was not performed, which should be considered a limitation of the cell-culture-based experimental model.

Future investigations should focus on elucidating the specific molecular cargo of these stress-induced EVs. In particular, the application of highly sensitive proteomic approaches will be essential to overcome the inherent challenge of low physiological EV protein yields and to identify specific protein patterns. Characterizing these functional molecules will further unravel the complex pathophysiology of pregnancy complications in dairy cattle.

## 5. Conclusions

In summary, we successfully isolated EVs from primary bovine caruncular epithelial cells. The isolated EVs fell within the expected size range for small EVs, and their composition included markers typical of small EVs. Furthermore, our in vitro model confirmed that exposure to cigarette smoke extract (CSE) significantly decreases cell viability and induces profound intracellular oxidative stress. Crucially, we demonstrated that this state of redox imbalance in maternal placental cells directly influences the quantity of vesicles released. Such an altered secretion of extracellular vesicles may indicate changes in the cellular response to oxidative stress at the foeto–maternal interface; however, its functional consequences for placental communication and foetal development require further investigation.

## Figures and Tables

**Table 1:** Size distribution and particle concentration of extracellular vesicles (EVs) across different size-exclusion chromatography (SEC) fractions. The analysis was performed using Tunable Resistive Pulse Sensing (TRPS). Normalized concentration was calculated by dividing TRPS-derived particle concentration by the number of viable cells present in the corresponding pooled sample after the 48 h EV-production phase and expressed per 10^6^ viable cells.

SEC Fraction	Parameter	Control (SM)	CSE 1%
Fractions 5–6	Particle diameter—mode (nm):	127	122
	Concentration (particles/mL)	1.79 × 10^9^	3.12 × 10^9^
	Normalized concentration (particles/mL/10^6^ viable cells)	6.16 × 10^7^	1.84 × 10^8^
Fractions 7–8	Particle diameter—mode (nm):	84	80
	Concentration (particles/mL)	4.48 × 10^10^	7.53 × 10^10^
	Normalized concentration (particles/mL/10^6^ viable cells)	1.52 × 10^9^	4.47 × 10^9^
Fractions 9–10	Particle diameter—mode (nm):	123	124
	Concentration (particles/mL)	1.87 × 10^8^	2.68 × 10^8^
	Normalized concentration (particles/mL/10^6^ viable cells)	6.41 × 10^6^	1.63 × 10^7^

## Data Availability

The original contributions presented in this study are included in the article and Supplementary Material. Further inquiries can be directed to the corresponding author.

## Supplementary Materials

The following supporting information can be downloaded at https://www.mdpi.com/article/10.3390/ani16111717/s1: Figure S1: Original Western blot image of CD9; Figure S2: Original Western blot image of flotillin-1.
