# Supplementary figures and images for: Effect of Oxidative Stress on Extracellular Vesicles Secreted by Caruncular Epithelial Cells Isolated from Bovine Placenta During Pregnancy—Preliminary Results
Source: Animals (Basel). 2026 Jun 4;16(11):1717. doi: 10.3390/ani16111717 (PMC13255872; doi:10.3390/ani16111717)

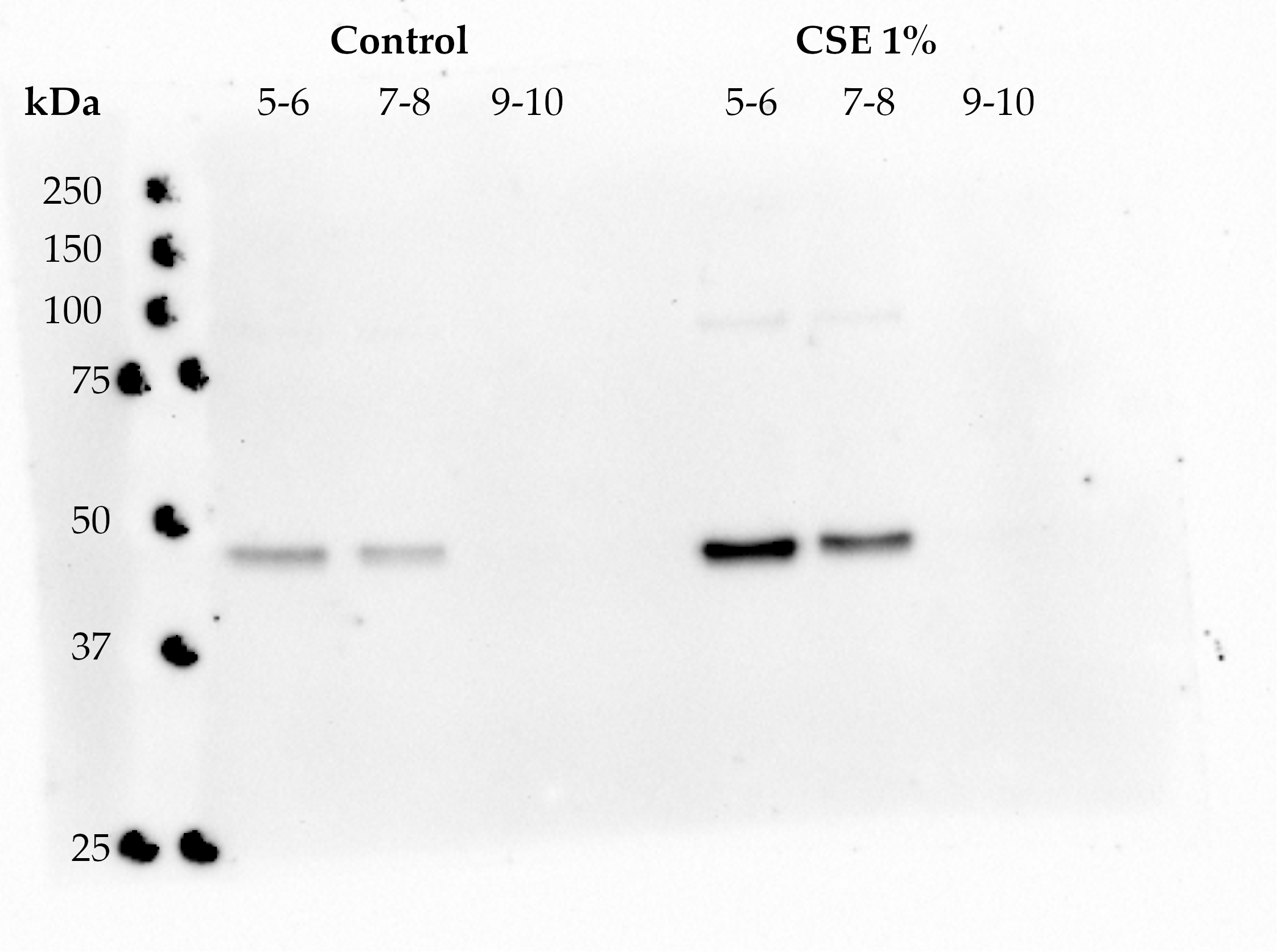

Supplement: Supplementary file 1 [file animals-16-01717-s001.zip › FigureS2_Flotillin-1.png]

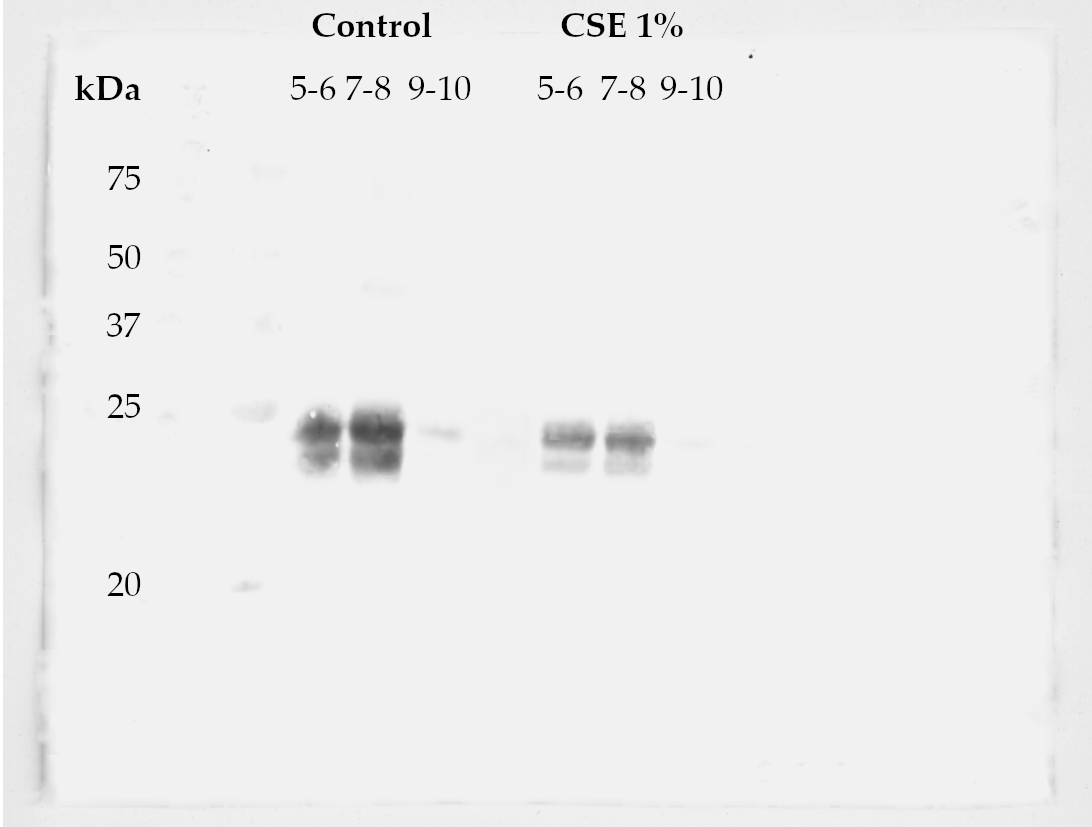

Supplement: Supplementary file 1 [file animals-16-01717-s001.zip › FigureS1_CD9.png]
